# Unravelling the Metabolic and Hormonal Machinery During Key Steps of Somatic Embryogenesis: A Case Study in Coffee

**DOI:** 10.3390/ijms20194665

**Published:** 2019-09-20

**Authors:** Rayan Awada, Claudine Campa, Estelle Gibault, Eveline Déchamp, Frédéric Georget, Maud Lepelley, Cécile Abdallah, Alexander Erban, Federico Martinez-Seidel, Joachim Kopka, Laurent Legendre, Sophie Léran, Geneviève Conéjéro, Jean-Luc Verdeil, Dominique Crouzillat, David Breton, Benoît Bertrand, Hervé Etienne

**Affiliations:** 1 Nestlé Research—Plant Science Unit, 101 avenue Gustave Eiffel, F-37097 Tours CEDEX 2, France; estelle.gibault@rdto.nestle.com (E.G.); maud.lepelley@rdto.nestle.com (M.L.); dominique.crouzillat@rdto.nestle.com (D.C.); david.breton@rdto.nestle.com (D.B.); 2 CIRAD (Centre de coopération internationale en recherche agronomique pour le développement), UMR IPME, F-34398 Montpellier, France; eveline.dechamp@ird.fr (E.D.); frederic.georget@cirad.fr (F.G.); sophie.leran@cirad.fr (S.L.); benoit.bertrand@cirad.fr (B.B.); herve.etienne@cirad.fr (H.E.); 3 UMR IPME (Interactions Plantes Microorganismes Environnement), University of Montpellier, CIRAD, IRD, F-34398 Montpellier, France; claudine.campa@ird.fr (C.C.); cecile.abdallah@ird.fr (C.A.); 4 IRD (Institut de recherche pour le développement), UMR IPME, F-34398 Montpellier, France; 5 Max Planck Institute for Molecular Plant Physiology, Am Muehlenberg 1, D-14476 Golm, Germany; erban@mpimp-golm.mpg.de (A.E.); mseidel@mpimp-golm.mpg.de (F.M.-S.); kopka@mpimp-golm.mpg.de (J.K.); 6 Université de Lyon (Université Lyon 1, CNRS, UMR5557, Ecologie Microbienne, INRA, UMR1418), F-69622 Lyon, France; laurent.legendre@univ-lyon1.fr; 7 Histocytology and Plant Cell Imaging platform PHIV, UMR AGAP (CIRAD, INRA, SupAgro)-UMR B&PMP (INRA, CNRS, SupAgro, University of Montpellier), F-34095 Montpellier, France; genevieve.conejero@inra.fr (G.C.); jean-luc.verdeil@cirad.fr (J.-L.V.)

**Keywords:** cell imaging, cell fate, coffee, histology, hormone content, metabolomics, somatic embryogenesis, totipotency

## Abstract

Somatic embryogenesis (SE) is one of the most promising processes for large-scale dissemination of elite varieties. However, for many plant species, optimizing SE protocols still relies on a trial-and-error approach. Using coffee as a model plant, we report here the first global analysis of metabolome and hormone dynamics aiming to unravel mechanisms regulating cell fate and totipotency. Sampling from leaf explant dedifferentiation until embryo development covered 15 key stages. An in-depth statistical analysis performed on 104 metabolites revealed that massive re-configuration of metabolic pathways induced SE. During initial dedifferentiation, a sharp decrease in phenolic compounds and caffeine levels was also observed while auxins, cytokinins and ethylene levels were at their highest. Totipotency reached its highest expression during the callus stages when a shut-off in hormonal and metabolic pathways related to sugar and energetic substance hydrolysis was evidenced. Abscisic acid, leucine, maltotriose, myo-inositol, proline, tricarboxylic acid cycle metabolites and zeatin appeared as key metabolic markers of the embryogenic capacity. Combining metabolomics with multiphoton microscopy led to the identification of chlorogenic acids as markers of embryo redifferentiation. The present analysis shows that metabolite fingerprints are signatures of cell fate and represent a starting point for optimizing SE protocols in a rational way.

## 1. Introduction

Somatic embryogenesis (SE) is a developmental process where a plant somatic cell can dedifferentiate to a totipotent embryogenic stem cell that has the ability to redifferentiate and give rise to an embryo under appropriate conditions [1,2,3]. This new embryo can further develop into a whole plant. Since its first description in carrot by Reinert [4] and Steward et al. [5], this process has been reported in a wide range of plant species, both annual [6,7,8] and perennial [9,10,11,12,13]. SE has demonstrated significant benefits when applied to forest tree species, bringing important advantages including clonal mass propagation, cryostorage of valuable germplasm and genetic transformation [14,15]. SE is particularly important for plants that have a long life cycle (woody species) and are difficult to propagate by conventional methods [16].

The ability of a somatic cell to undergo embryogenesis in vitro is both an inherent and an acquired characteristic that requires the right combination of explant and culture environment [17]. The most efficient treatments used to induce embryogenesis are diverse and range from application of exogenous growth regulators to abiotic stress. Under the appropriate conditions, the explant produces histo-differentiated embryos, either directly from the explant or indirectly from callus [18]. The direct SE is often described as a low yield method and the indirect SE as a high yield method [10]. The morphological and cellular changes that occur during in vitro embryogenesis have been well-described [1,19,20]. Briefly, nine developmental stages have been characterized in the indirect SE of dicots: explant, primary callus, embryogenic callus, embryogenic cell clusters, pro-embryogenic masses, globular-shaped embryos, heart-shaped embryos, torpedo-shaped embryos and cotyledonary embryos, before development into a new plant [1,19,20]. Unlike the detailed knowledge about morphological and histological events, little is known about the underlying mechanisms involved in the acquisition and expression of totipotency occurring during SE.

Although SE has already been widely described in a number of woody species [10,11,12,13] propagating adult woody plants remains a difficult, laborious, intensive and tricky operation. Many authors reported that the lack of knowledge on the mechanisms underlying the reprogramming of somatic cells represents the greatest limitation to the improvement of the SE process [21,22]. Research on SE remains mainly empirical, characterized by a low-throughput trial-and-error approach. A set of drawbacks have been reported especially a strong genotypic effect, a difficulty in obtaining embryogenic callus, a variability in the quality of regenerated embryos, and more generally a lack of efficiency of certain steps [9,23,24] leading to hitherto prohibitive production costs and an overall slow technical progress.

In parallel, the latest omics technologies (genomics, transcriptomics, proteomics, epigenomics, metabolomics and phenomics) are gaining increasing attention. Many authors believe that combining omics technologies with the in vitro process can have a tremendous impact on the knowledge of the molecular mechanisms underlying SE [22,25,26]. Metabolomics data can provide a wealth of information in the description and elucidation of physiological responses to environmental conditions in plants [27]. However, to date, very few authors have carried out a metabolomics approach related to SE. Using gas chromatography–mass spectrometry (GC-MS), metabolomic profiling has been successfully applied to identify metabolic processes regulating SE in five sampled stages in Norway spruce (*Picea abies*) covering cell line proliferation till embryo maturation [28]. Similarly, Robinson et al. [29] applied a GC-MS approach in loblolly pine (*Pinus taeda*) as a way to predict a model for embryo regeneration capacity. More recently, Dobrowolska et al. [30] applied GC-MS to compare metabolic profiles of normal and aberrant SE germinants in Norway spruce. In the same species, using liquid chromatography-mass spectrometry (LC-MS), Vondrakova et al. [31] drew endogenous phytohormone profiles in nine sampled stages covering proliferation, maturation, desiccation and germination. Using the same technique, Gautier et al. [32] compared phytohormone profiles between embryogenic calli and non-embryogenic calli in Douglas-fir (*Pseudotsuga menziesii)*. All these authors assumed that clear correlations may exist among the different metabolome or phytohormone profiles and some specific SE stages. Nonetheless, to date, no global analysis, covering leaf explant dedifferentiation till embryo development, of both metabolome and hormone content has been performed on a complete SE process.

Coffee is one of the world’s favorite beverages. It has a great economic impact in many producing countries, especially in South America [33]. Today, SE applied to coffee is one of the most advanced technologies in plant mass vegetative propagation [24]. Thirty years of research on SE by two leading groups—the CIRAD/ECOM alliance and Nestlé—has led to a successful large-scale dissemination of *Coffea arabica* F1 hybrids and *C. canephora* cv. Robusta clones for 13 years now. Even though good biological efficiencies characterize the processes established in the two cultivated species, with low genotypic effects [34,35] and controlled somaclonal variation [36,37], SE production cost remains high and still cannot compete with production costs of seedlings or traditional cuttings [38].

Indeed, as for other species, coffee SE research remains mainly empirical, also characterized by overall slow technical progress. For example, the development of culture conditions for the mass redifferentiation into somatic embryos have been very long (10 years) and laborious, carried out only by an empirical approach [24]. The lack of knowledge about the cellular and molecular events taking place during these developmental stages makes these stages real black boxes. While a scale-up is needed to meet increasing market demand—estimated at 50–100 million coffee vitroplants per—current overall production cannot meet this huge demand [24]. However, optimization is possible for different stages if a rational optimization is developed on metabolic knowledge.

In this paper, we describe how we took advantage of the latest omics technologies combined with a reliable, synchronized and efficient large-scale coffee SE process to draw full metabolic profiles and study hormone dynamics over 15 key sampled stages. A solid statistical method was used to identify the metabolic pathway changes associated with the main developmental phases and phase switches. Histological analysis and cell imaging were also required to characterize sampled stages and associate metabolic profiles with cell structure organization. Lastly, comparing Arabica embryogenic and non-embryogenic calli enabled the identification of metabolic markers of the embryogenic capacity.

## 2. Results

### 2.1. Morphological and Histological Characterization of Arabica SE Key Sampled Stages

**Figure 1:**
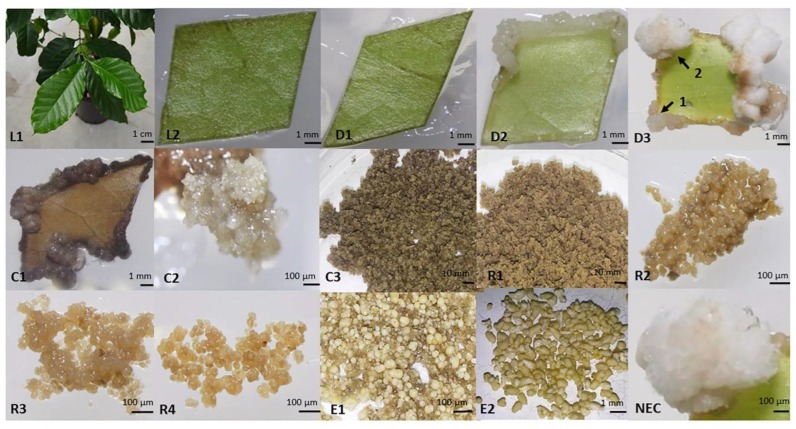
Collection of samples from leaf explant till embryo development covering 14 key sampled stages of the Arabica SE process: leaves from greenhouse plants (L1), explants during dedifferentiation [0 h (L2), 1 week (D1), 2 weeks (D2), 5 weeks (D3)], compact primary callus obtained 3 months after induction (C1), embryogenic callus obtained 7 months after induction (C2), established cell clusters obtained after 4 months in proliferation medium (C3), early redifferentiation from cell clusters [1 week in DIF medium without reducing cell density (R1), 24 h in DIF medium after reducing cell density (R2), 72 h (R3), 10 d (R4)], globular embryos (E1) and torpedo-shaped embryos (E2). Non-embryogenic callus (NEC) was also sampled at the same time as the embryogenic callus (C2). Images were taken using an Olympus E-5 digital camera mounted on an Olympus SZX7 stereomicroscope. The stereomicroscope was not used for (L1). Two types of calli can be seen in (D3): an organized yellowish callus (1) and a whitish spongy callus (2).

**Figure 2:**
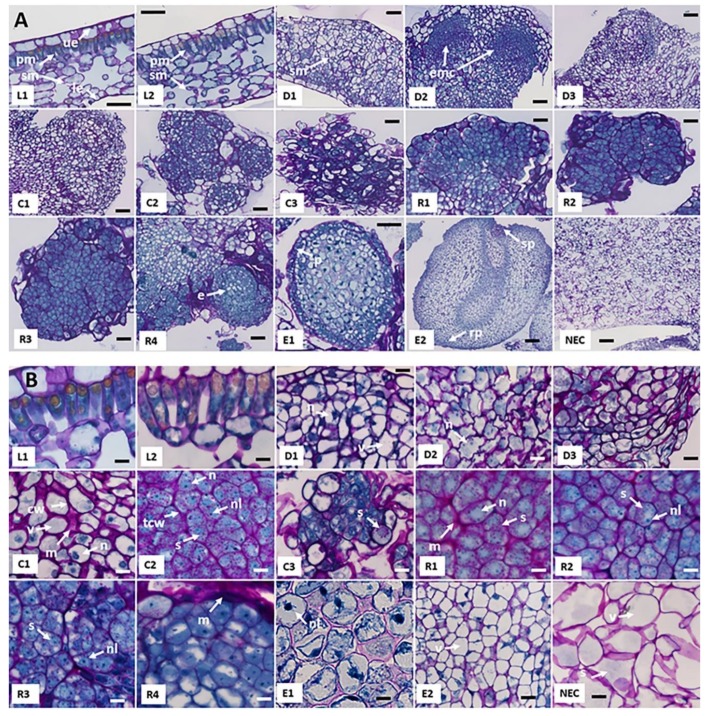
Characterization of 15 key sampled stages throughout the Arabica SE process at a histological (**A**) and cellular level (**B**). Sampled stages correspond to: leaves from greenhouse plants (L1), explants during dedifferentiation [0 h (L2), 1 week (D1), 2 weeks (D2), 5 weeks (D3)], compact primary callus obtained 3 months after induction (C1), embryogenic callus obtained 7 months after induction (C2), established cell clusters obtained after 4 months in liquid proliferation medium (C3), pro-embryogenic masses [1 week in redifferentiation medium after auxin withdrawal (R1), 24 h in redifferentiation medium after reducing cell density (R2), 72 h (R3), 10 days (R4)], globular embryos (E1) and torpedo-shaped embryos (E2). An additional stage, the non-embryogenic callus (NEC), was also sampled at the same time as the embryogenic callus (C2) and obtained in the same culture conditions. Sections were treated with 1% periodic acid and stained with Schiff reagent (colors polysaccharides in purple) and Naphthol Blue Black (colors soluble proteins in blue). Images were taken in bright field. The scale bar is set to 50 µm in (**A**) and 10 µm in (**B**). White arrows indicate: cw: cell wall, e: embryoid structure, emc: emerging callus, le: lower epidermis, m: mucilaginous coating, n: nucleus, nl: nucleolus, p: protodermis, pm: palisade mesophyll cells, rp: root pole, s: starch, sm: spongy mesophyll cells, sp: shoot pole, tcw: thickened outer cell wall, ue: upper-epidermis, v: vacuole.

A characterization of the 15 key sampled stages of the Arabica SE process on a morphological level (Figure 1), as well as on a histocytological level is shown in Figure 2 and Table 1. SE was carried out from juvenile leaf explants (Figure 1L2) which could be characterized histologically by a well-structured anatomy i.e., from up to bottom, an upper-epidermis, palisade mesophyll cells, spongy mesophyll cells and a lower-epidermis (Figure 2A L1). Palisade mesophyll cells are elongated and cuboid (width-to-length ratio = 0.33 ± 0.04) (Figure 2B L1, Table 1) with a low nucleocytoplasmic (N/C) ratio (0.17 ± 0.02) and a small nucleus located at the periphery of the cell, pressed against the cell wall (Figure 2B L1). Leaf disinfection (Figure 2B L2) did not have a significant effect on cell morphology. After one week on culture medium, and even though no changes were noticed at a morphological level (Figure 1D1), a high mitotic activity was taking place in cells enduring dedifferentiation and evidenced by the number of cells undergoing cytokinesis (Figure 2B D1). This mitotic activity was particularly observed in perivascular and spongy mesophyll cells of the leaf explant. Cell nuclei appeared intensely stained in blue by Naphthol Blue-Black and took a more or less central position, where they were maintained by spans of cytoplasm between which are found several vacuoles of small sizes. Cells were therefore described as star-shaped. These vacuolated cells had a large size (647 ± 71 µm^2^) and a high width-to-length ratio (0.67 ± 0.08) (Table 1). After two weeks on culture medium, whitish amorphous callus started to emerge from the wounded edge of leaf explants (Figure 1D2). On a histological level, clusters of actively proliferating meristematic cells appeared to be forming the emerging callus (Figure 2A D2). These cells were remarkably stained with Naphthol Blue Black. Dedifferentiated cells of the constituted callus had an irregular shape, but maintained their large size and high mitotic activity as well as a higher N/C ratio (2.7-fold) (Figure 2B D2, Table 1). After five weeks on culture medium, two types of calli could be observed: a whitish spongy callus which is non-embryogenic and a more compact and organized yellowish callus that emerged on the edge of leaf explants (Figure 1D3). The cells from the yellowish callus were also actively proliferating (numerous mitosis) with a larger size (695 ± 91 µm^2^) (Figure 2B D3, Table 1). Three months after the in vitro introduction, this callus was well-established and had a dark grey color, and named primary callus (Figure 1C1 and Figure 2A C1). Primary callus cells were square-shaped, had also a large size (784 ± 56 µm^2^), a large nucleus (114 ± 25.3 µm^2^), and their vacuole occupied the main cell volume (Figure 2B C1, Table 1). A polysaccharide sugar-containing mucilaginous coating layer was present between cell walls (Figure 2B C1). Less cell divisions were observed in this type of slow proliferating callus. Seven months after explant introduction, embryogenic calli emerged (Figure 1C2 and Figure 2A C2). Embryogenic calli were yellowish and friable, constituted by small cells (214 ± 18 µm^2^), isodiametric, surrounded by a thickened outer cell wall, arranged in clusters, with a dense cytoplasm (rich protein-staining) and a central nucleus with a prominent nucleolus stained in blue-black (N/C ratio = 0.33 ± 0.02) (Figure 2B C2, Table 1). The nucleus is surrounded by starch granules. These cells display the characteristic cytological features generally found in embryogenic calli [39]. A morphological and histological comparison between embryogenic and non-embryogenic calli (NEC) is described below. Embryogenic cell characteristics were maintained in established embryogenic cell clusters (Figure 2B C3) with a sharp increase in the number of proliferating cells. Indeed, cell clusters were maintained for four months in proliferation medium and became brownish in color (Figure 1C3). All pro-embryogenic masses (PEMs) collected during redifferentiation (Figure 2B R1, R2, R3, R4) shared the same characteristics with embryogenic cell clusters on histological and cellular levels with a higher ratio of square-shaped cells (width-to-length ratio = 0.70 ± 0.07 vs. 0.54 ± 0.06, Table 1). Easily observable, cell cluster dark brown color quickly turned to light brown as soon as the redifferentiation step was induced (Figure 1C3,R1). A sugar-containing mucilaginous coating layer was present between cell walls (Figure 2B R1). The formation of globular embryo structures could be seen from the R4 stage in which a sugar mucilaginous coating layer was backing the newly formed embryoids (Figure 2A R4). While PEMs were characterized by a high N/C ratio, embryonic cells of globular-shaped embryos (Figure 2A E1) were characterized by a small nucleus hence a low N/C ratio (0.10 ± 0.01 vs. 0.31 ± 0.03, Table 1) and a dense nucleus at the edge of the cell with the vacuole occupying the main cell volume (Figure 2B E1). This aspect was more highlighted in embryonic cells of the ground promeristem of torpedo-shaped embryos (Figure 2A E2) where cells were circular and of larger size (943 ± 55 µm^2^) (Figure 2B E2). Globular and torpedo-shaped embryos were white in color (Figure 1E1,E2). Globular embryos were surrounded by a protodermis (outer layer) (Figure 2A E1). Torpedo-shaped embryos were bipolar structures with easily distinguishable shoot and root poles (Figure 2A E2).

### 2.2. Clustering Metabolite Profiles Divides the Arabica SE Process into Five Major Phases

**Figure 3:**
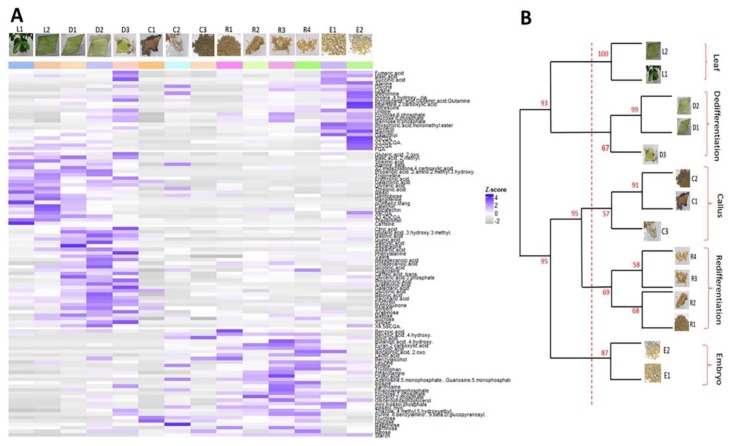
Profiling primary and secondary metabolites during 14 key sampled stages of the Arabica somatic embryogenesis process. (**A**) Heatmap generated from the Z-score values of a total of 92 primary metabolites and 12 secondary metabolites detected. Rows correspond to metabolites and columns to the sampled stages. Positive Z-score values are shown in blue and negative values in grey. (**B**) Hierarchical clustering of the 14 sampled stages according to the similarities in their metabolic profiles. The clustering was performed using Pearson’s correlation coefficient. Cluster probabilities were calculated via a multiscale bootstrap with a total of 1000 iterations. Clustering yielded 5 major nodes: Leaf, Dedifferentiation, Callus, Redifferentiation and Embryo.

A total of 92 primary metabolites and 12 secondary metabolites were detected over the 14 SE sampled stages (Table S1). The heatmap generated from the Z-score values of the total primary and secondary metabolites detected showed similar patterns in some of these stages (Figure 3A). We decided to carry out a hierarchical clustering analysis to highlight stages that shared similar metabolic patterns. Strong correlations between patterns were obtained as the threshold applied (red line, Figure 3B) yielded 5 major nodes: the “Leaf” node where the L1 and L2 stages clustered together (cluster probability = 100%), the “Dedifferentiation” node where the D1, D2 and D3 stages clustered together (cluster probability = 67%), the “Callus” node where the C1, C2 and C3 stages clustered together (cluster probability = 57%), the “Redifferentiation” node where the R1, R2, R3 and R4 stages clustered together (cluster probability = 69%) and the “Embryo” node where the E1 and E2 stages clustered together (cluster probability = 87%). The obtained clusters grouped along the axis of the SE process as the obtained nodes corresponded to successive developmental phases of the SE process, i.e., the leaf phase, the leaf explant dedifferentiation phase, the callus phase, the redifferentiation phase (embryogenic cell clusters to embryoid structures), and the embryo development phase (globular to torpedo-shaped embryos).

### 2.3. Metabolic Characterization of the Five Phases of the Arabica SE Process

For each developmental phase, primary and secondary metabolites displaying significant increased values from their respective means across the 14 stages (Z-score > 1) are listed in Table 2 and those displaying significant decreased values (Z-score < −1) are listed in Table 3. The “Leaf” phase highlighted an accumulation of two amino acids (beta-alanine, aspartic acid), metabolites implicated in the ascorbate and aldarate metabolism (galactonic and threonic acid) and also primary metabolites involved in the shikimate pathway (shikimic and quinic acid), as well as secondary metabolites production such as alkaloids (trigonelline, theobromine and caffeine) and phenolic compounds (mangiferin, catechin, epicatechin, 3-caffeoylquinic acid (3-CQA), 4-CQA, 3,4-diCQA, feruloylquinic acid (FQA)). The “Leaf” phase is also characterized by a decrease of sugars (fructose, ribose) and phosphorylated sugars (glucose-6-phosphate, fructose-6-phosphate, mannose-6-phosphate), two amino acids (leucine, lysine) and myo-inositol.

The “Dedifferentiation” phase showed an accumulation of metabolites involved in the tricarboxylic acid (TCA) cycle (aconitate, 2-oxoglutarate, succinate, fumarate, malate, citrate) and in the ascorbate and aldarate metabolism (arabinonic acid, galatactaric acid, galactonic acid). It also included an accumulation of sugars (maltose, sucrose), phosphorylated sugars (glucose-6-phosphate, fructose-6-phosphate, mannose-6-phosphate), amino acids (beta-alanine, aspartic acid, asparagine, glycine, glutamine, valine), and fatty acids (hexadecanoic acid, octadecanoic acid, glyceric acid). Primary metabolites involved in secondary metabolism pathways were also accumulated such as phenylalanine, caffeic and quinic acid, nicotinic acid and xanthosine. During this phase, lower levels were obtained for some carbohydrates such as glucose, fructose and starch and one amino acid, lysine.

The “Callus” phase displayed an accumulation of some sugars (maltotriose, ribose, glucose, fructose), amino acids (glycine, methionine, proline), fatty acids (glycerol), and myo-inositol. This phase induced a decrease for metabolites implicated in the TCA cycle and the ascorbate and aldarate metabolisms, as well as for phosphorylated sugars, sucrose and three amino acids (leucine, lysine, valine).

The “Redifferentiation” phase showed an accumulation of phosphorylated sugars, ribose, three amino acids (leucine, lysine, proline), glycerol-3-phosphate, myo-inositol, and aconitate. Primary metabolites involved in secondary metabolism pathways were also accumulated such as tryptophan, caffeic and benzoic acid, guanosine, inosine and xanthosine. Only valine exhibited a significant decrease for this stage.

The “Embryo” phase (globular to torpedo-shaped embryos) included an accumulation of TCA cycle metabolites, phosphorylated sugars, starch, amino acids (alanine, glycine, leucine, lysine, methionine, proline, glutamine, tryptophan), polyols (myo-inositol, mannitol), glycerol, glycerol-3-phosphate and putrescine. Phenolic compounds (3-CQA, 4-CQA, 5-CQA, 3,4-diCQA, 3,5-diCQA, FQA) were accumulated again, as in the “Leaf” phase. Only fructose was decreased.

**Figure 4:**
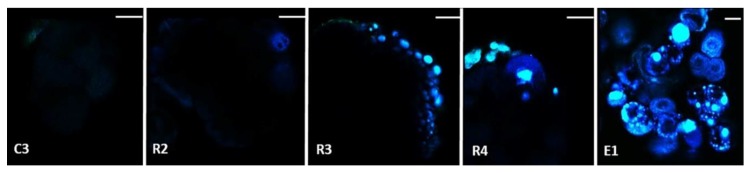
Chlorogenic acid localization in embryogenic cell clusters (**C3**) and during embryo redifferentiation after 1 day (**R2**), 3 days (**R3**), 10 days (**R4**) and 3 weeks (**E1**) in redifferentiation medium after cell density reduction. Protuberance of newly generated embryos emerge after 10 days in redifferentiation medium and globular-shaped embryos are obtained after 3 weeks. For chlorogenic acid localization, fresh samples were mounted on a glass slide in a drop of water and observed using a Zeiss LSM880 multiphoton microscope, equipped with a Chameleon Ultra II laser. Excitation wavelength was set to 720 nm and emission was observed with a band-pass filter 386–502 nm (blue). The presence and accumulation of total chlorogenic acids during embryo redifferentiation was confirmed by multiphoton microscopy combined with emission spectral analysis of total chlorogenic acids (Figure S2). The scale bar is set to 100 µm.

The presence and accumulation of total chlorogenic acids during embryo redifferentiation was confirmed by multiphoton microscopy combined with emission spectral analysis of total chlorogenic acids in PEMs after 1 day (R2), 3 days (R3), 10 days (R4) and 3 weeks (E1) in redifferentiation medium (Figure 4 and Figure S2). Emission spectral analysis allows to specifically associate emission spectra from cells with defined pure autofluorescent compounds (in this case, total chlorogenic acids) [40]. Absent in embryogenic cell clusters, total chlorogenic acids seemed to accumulate on the edge of PEMs at day 3 at the same location where the embryo structure later emerged (at day 10). Autofluorescence intensity of total chlorogenic acids was strongly associated with the number of globular-shaped embryos obtained after 3 weeks. Taken together, these findings indicate that chlorogenic acids are potent metabolic markers of the embryonic state.

### 2.4. Cell Hormonal Content Changes throughout the Arabica SE Process

Analysis of cell hormonal content showed variations over the five developmental phases of the SE process (Table 4). When switching from the “Leaf” phase to the “Dedifferentiation” phase, indole-3-acetic acid (IAA), 1-aminocyclopropane-1-carboxylic acid (ACC) and zeatin (*cis*- and *trans*-) (Z) levels significantly increased whereas *cis*-abscisic acid (ABA) levels significantly decreased. Moreover, indole 3-butyric acid (IBA) and isopentenyladenine (iP) levels were only detectable in the “Dedifferentiation” phase. ABA glucose ester (ABAGE) and zeatin riboside (*cis-* and *trans*-) (ZR) levels were unaffected.

When switching from the “Dedifferentiation” phase to the “Callus” phase, IAA, ACC and Z levels significantly decreased while ABAGE and ZR levels significantly increased. ABA was maintained at similar levels.

When switching from the “Callus” phase to the “Redifferentiation” phase, IAA levels increased again whereas ZR levels dropped. ABA, ABAGE, ACC and Z were maintained at similar levels.

When switching from the “Redifferentiation” phase to the “Embryo” phase, ABA, ABAGE, Z and ZR levels significantly increased. IAA and ACC levels were unaffected.

### 2.5. Most Significant Changes in Metabolic and Hormonal Profiles Occur during Phase Switches

**Figure 5:**
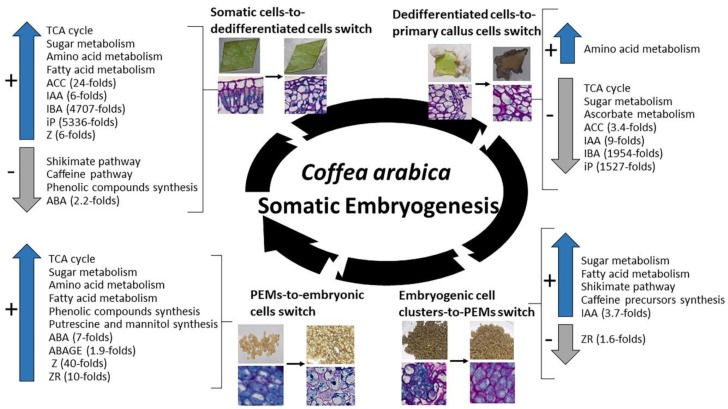
Metabolic pathways and hormone dynamics during the four main developmental phase switches. The four phase switches are: the leaf explant-to-dedifferentiated cell switch occurring after one week in induction medium, the dedifferentiated cells-to-established primary callus cells switch occurring three months after induction, the embryogenic cell clusters-to-pro-embryogenic masses (PEMs) switch occurring after one week in redifferentiation medium, and the PEMs-to-embryonic cells switch occurring after four weeks in redifferentiation medium. Metabolic pathways were identified based on an enrichment in significantly over-accumulated and under-accumulated metabolites, i.e., Z-score > 1 and Z-score < −1, respectively. All presented hormones differ significantly in their levels according to a two-by-two Wilcoxon mean comparison test (*P* < 0.05). Values represent mean of ratios in hormone levels of independent replicates between a sampled stage and the previous one ± SD.

A developmental phase switch corresponds to the transition between the last sampled stage of a developmental phase and the first sampled stage from the following phase. Since five main phases were highlighted (Leaf, Dedifferentiation, Callus, Redifferentiation, Embryo), four phase switches could be identified and summarized in Figure 5:

(i) The leaf explant-to-dedifferentiated cell switch occurring after one week in induction medium (L2 stage to D1). During this switch, and even though no change could be observed on a morphological basis, huge changes were noticed at a biochemical level. Our results show a drastic increase in levels of metabolites involved in the TCA cycle pathway, in sugar (glucose-6-phosphate, fructose-6-phosphate, mannose-6-phosphate), amino acid and fatty acid metabolisms while shikimate, caffeine and phenolic compounds pathways were poorly present. Hormone levels also sharply increased during this phase switch mainly ACC (24-fold), IAA (6-fold), IBA (4707-fold), iP (5336-fold) and Z (6-fold), except for ABA which levels were decreased (2.2-fold).
(ii) The dedifferentiated cells-to-established primary callus cells switch occurring three months after induction (D3 stage to C1). This switch was characterized by a sharp decrease in cell basic functions, mainly a decrease in the levels of metabolites involved in the TCA cycle pathway, sugar metabolism (phosphorylated glucose and fructose), and the ascorbate pathway which is related to the cell photosynthetic activity. Only synthesis of some amino acids was maintained (methionine, glycine). Hormone levels also sharply decreased in compact primary callus cells compared to cells in early dedifferentiation mainly IBA (1954-folds), iP (1527-fold), IAA (9-fold) and ACC (3.4-fold).
(iii) The embryogenic cell clusters-to-PEMs switch occurring after one week in redifferentiation medium (C3 stage to R1). During this switch, a re-activation in cell primary and secondary functions was observed; levels of phosphorylated sugars (glucose-6-phosphate, fructose-6-phosphate, mannose-6-phosphate) and fatty acids (glycerol-3-phosphate) increased as well as precursors of caffeine (inosine, xanthosine) and end-products of the shikimate pathway (caffeic acid, benzoic acid, tryptophan). In parallel, IAA levels also increased in PEMs compared to embryogenic cell clusters (3.7-fold) while levels of ZR sharply decreased (1.6-fold).
(iv) The PEMs-to-embryonic cells switch occurring after four weeks in redifferentiation medium (R4 stage to E1). During this switch, cell functions are re-established in embryonic cells with increased levels of TCA cycle metabolites, phosphorylated sugars and fatty acids, and the synthesis of a number of amino acids (alanine, glycine, leucine, lysine, methionine, proline, glutamate) and phenolic compounds (5-CQA; 3-CQA; 4-CQA; 3,5-di-CQA; 4,5-diCQA and FQA). A synthesis of putrescine and mannitol was also noticed. Our results also showed a significant increase in ABA levels (7-fold), ABAGE levels (1.9-fold), Z levels (40-fold) and ZR levels (40-fold) in embryonic cells compared to PEMs.

These results clearly show that comparing metabolic profiles between two sampled stages corresponding to a phase switch allowed obtaining a better description of these stages than a morphological or histological comparison as it made possible to distinguish between two sampled stages that are morphologically similar (e.g., leaf explant before and after one week on dedifferentiation medium) or histologically similar (e.g., dedifferentiated cells and established primary callus cells).

### 2.6. Embryogenic and Non-embryogenic Calli Differ Morphologically, Metabolically and Hormonally

Embryogenic (EC) and non-embryogenic calli (NEC) can be easily distinguished on the basis of their morphology, color and cellular characteristics (Figure 2A C2, NEC; Table 5). Somatic embryos could not be regenerated from NEC. EC developed from compact primary calli whereas NEC developed from non-compact primary calli. EC were yellowish and friable, constituted by small cells (214 ± 18 µm^2^), isodiametric, surrounded by a thickened outer cell wall, arranged in clusters, with a dense cytoplasm (rich protein-staining) and a central nucleus with a prominent nucleolus stained in blue-black (N/C ratio = 0.33 ± 0.02) (Figure 1C2 and Figure 2B C2). NEC were spongy and translucent, displaying cells of a much larger size (2081 ± 386 µm^2^) with the vacuole occupying a big share of the cytoplasm, and an absence of cytoplasmic organelles (Figure 1 NEC and Figure 2B NEC). The nucleocytoplasmic ratio in NEC was significantly lower than in EC (0.17 ± 0.02 vs 0.33 ± 0.02). Protein and sugar staining-surfaces were limited to cell membranes and cell walls. A characteristic attribute of EC in coffee was the abundance of starch granules whereas larger insoluble starch reserves were found in NEC.

**Figure 6:**
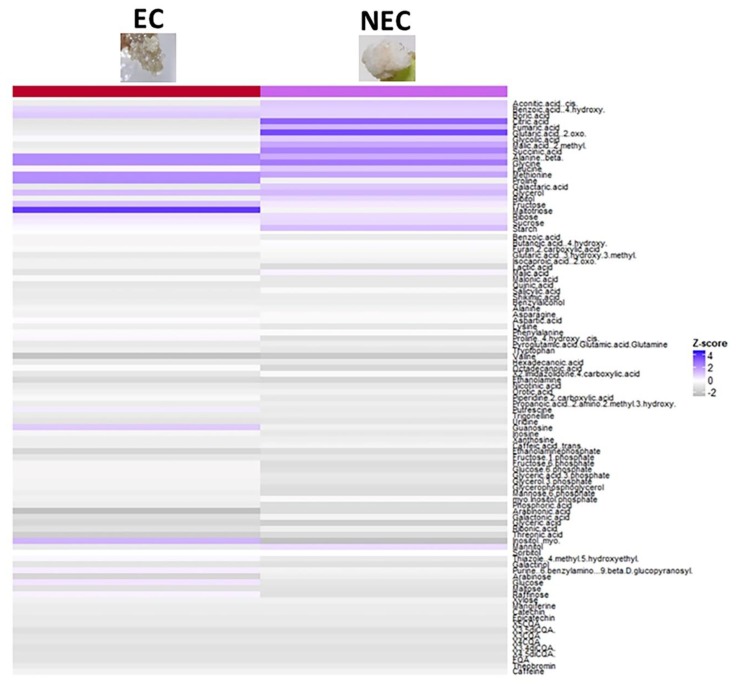
Profiling primary and secondary metabolites in embryogenic (EC) and non-embryogenic calli (NEC). Heatmap generated from the Z-score values of a total of 92 primary metabolites and 12 secondary metabolite detected. Rows correspond to metabolites and columns to the type of callus. Positive Z-score values are shown in blue and negative values in grey.

At a biochemical level, the heatmap generated from primary and secondary metabolite profiles of both tissues (Figure 6) displayed significantly increased and decreased levels for some metabolites (Z-score > 1 and Z-score < −1, respectively) listed in Table 6. Both EC and NEC accumulated ribose, amino acids (methionine and glycine) and fatty acids (glycerol) (Table 6) and had negative Z-scores for phosphorylated sugars, metabolites related to ascorbate and aldarate pathways and valine. However, while NEC over-accumulated TCA cycle metabolites and two amino acids (β-alanine, lysine) and under-accumulated a polyol (myo-inositol) and an amino acid (proline), EC over-accumulated sugars (glucose, fructose, maltotriose), an amino acid (proline), myo-inositol, and under-accumulated TCA cycle metabolites, an amino acid (leucine) and sucrose.

Concerning hormones, both EC and NEC had similar levels of ACC [2855-3177 ng/g DW] and IAA [19–32 ng/g DW] (Table 7). However, levels of ABA and ABAGE were significantly higher in EC than in NEC (16- and 50-fold, respectively), as well as the levels of ZR (4-fold). Z was only detected in EC (2 ± 1 ng/g DW). IBA and iP were not detected in either tissues.

## 3. Discussion

### 3.1. Arabica SE Metabolites as a Signature of Cell Fate

This study reports the first global analysis of SE metabolome and hormone dynamics, conducted on 15 key sampled stages covering the entire SE process, from leaf explant dedifferentiation to embryo development. Two pre-requisites were mandatory for this study: (i) the availability of large-scale protocols for coffee SE, offering high reliability and cell synchronicity in each sampled stage as well as a good efficiency; (ii) the availability of the latest omics technologies allowing to rapidly offer masses of information. This first global analysis applied on a model tree species for SE should serve as a reference for a wide range of plant species as the detailed sampling conducted on 15 stages provided a better field-of-view to the SE process as a whole and enabled to clarify some real black boxes [24]. The statistical approach employed in this study helped to clearly divide the SE process into five phases and four key phase switches that are strategic for the whole biological efficiency of embryo redifferentiation. Many authors recently reported the necessity of a better understanding of the SE process to clarify the current bottlenecks [22,26,41]. The fact that each of the metabolic profiles correlated with a specific cell status—leaf cells, cells in dedifferentiation, callus cells, pro-embryogenic masses, embryonic cells—is extremely relevant. This shows that metabolite fingerprints are a specific signature for each developmental phase and bring rich information on cell status, as some of these stages were undistinguishable by morphological and histological approaches. A number of papers on conifers assumed that clear correlations may exist among the different metabolome or phytohormone profiles and some specific SE stages (cell line proliferation to embryo maturation) [28,29,31]. This global analysis is a proof that metabolites could be good predictors of all cell fate transitions—from leaf explant introduction until embryo development—and could be used to control the SE process key stages. This goes beyond describing cell morphology and color, which was until now the only way to support empirical protocol optimization. Combining this study with transcriptomic approaches could give a much clearer understanding of the molecular mechanisms underlying cell reprogramming [22].

### 3.2. The Dedifferentiation Episode Is Characterized by Huge Changes in Cell Metabolic Pathways

SE is a process driven by exogenously supplied plant growth regulators. Although most plants require similar physical conditions (temperature, light regime) for the induction of SE, medium composition can have a large impact on SE outcome. According to Sugimoto et al. [42], the prime characteristic of plant regeneration is cell fate reprogramming induced by wounding, stress, and hormones. Auxins and cytokinins are widely known to play essential roles in the induction of embryogenic cultures [31]. The combined addition of natural (IBA and N^6^-(2-Isopentenyl) adenine (2-iP)) and synthetic (2,4-dichlorophenoxyacetic acid (2,4-D) and 6-benzylaminopurine (6-BA)) as exogenous growth regulators in the medium sharply increased endogenous levels of IAA and Z. The synthesis of endogenous auxins is considered as a crucial early step in the switch to totipotent growth. The results reported by different authors [43,44] account for an important role of auxins in SE induction. The precursor of ethylene, ACC, also showed a sharp increase during cellular dedifferentiation in our study and has been reported to improve somatic embryo induction in *Arabidopsis thaliana* [45].

Somatic cells within the plant contain all the genetic information necessary to create a complete and functional plant (with the exception of anuclear vascular cells) [6]. During cellular dedifferentiation, the existing developmental information of somatic cells must be switched off or re-configured to make the somatic cells ready for an embryogenic program [46]. Our results showed that secondary metabolites (phenolic compounds and alkaloids) no longer accumulated during this stage, whereas their precursor levels increased (phenylalanine and caffeic acid; nicotinic acid and xanthosine, respectively). Nic-Can et al. [47] have published a demonstration on the effect of caffeine and chlorogenic acid in SE of *C. canephora*. These authors have provided evidence that those compounds inhibited the embryogenic process by affecting DNA methylation. Since leaf explants are placed in the dark, metabolite routes that are dependent on the photosynthetic activity such as the ascorbate metabolism are slowly replaced by cell respiratory pathways, mainly glycolysis and TCA cycle. Glycolysis is a key and ubiquitous metabolic pathway that allows plant cells to convert carbohydrates into the energetic coin ATP. Our results are in agreement with a number of studies that showed that the glycolytic pathway is used by cells undergoing dedifferentiation [48,49].

Callus induction is a spectacular phenomenon in which important changes in tissue structure take place. Xu et al. [50] showed that genes related to cell wall hydrolysis and lipid metabolism are quickly activated after auxin treatment. Our results revealed an accumulation of amino acids and lipid residues that could originate from the fast degradation of membranes and proteins.

### 3.3. Identifying Metabolic Markers of the Embryogenic Capacity

To date, many authors have distinguished EC from NEC based on morphological and histological studies [51,52,53]. Biochemical comparison of these tissues showed that carbohydrates, phytohormones and free amino acids differentially accumulated in EC and NEC [32,54,55]. When comparing metabolomic and hormonal profiles of EC and NEC, we were able to identify metabolic markers of the embryogenic state that can be directly used to pilot the optimization of the composition of the culture medium. An over-accumulation of maltotriose, myo-inositol, proline, ABA and Z was characteristic of EC as opposed to NEC. On the contrary, an under-accumulation of leucine and TCA cycle metabolites was found in EC NEC. These metabolites could serve as predictors of the regenerative capacity.

As observed through histological analysis, EC is characterized by the abundance of starch granules, whereas larger insoluble starch reserves were found in NEC. Metabolite profiles in our study showed that NEC accumulated starch reserves, while EC highly accumulated maltotriose, a product of starch hydrolysis. Gautier et al. [32] also observed large structures of starch reserves in NEC of Douglas-fir (*P. menziesii*) and hypothesized that NEC main fate was survival while EC was a transient state before fate transition. We highlight here for the first time that maltotriose is the preferred way to store sugars in EC for a faster usage during fate transition. Other metabolic markers of the embryogenic state, myo-inositol and proline, that were abundant in EC, were detected at low levels in NEC. High levels of inositol were also found in Norway spruce cell lines competent for SE and are involved in stress responses enabling embryo formation [56]. Guillou et al. [57] reported a better embryo-to-plant conversion in *Theobroma cacao* with medium supplemented with myo-inositol. Liang et al. [58] pointed out that proline metabolism stimulates cell-signaling pathways through increased formation of reactive oxygen species (ROS) that promote embryo formation. Neves et al. [54] also found an accumulation of free proline in EC cells of sugarcane. Another metabolic marker of the embryogenic state was leucine which levels were severely lower in EC than in NEC. This could be due to the synthesis of leucine-rich repeat (LRR) proteins which are fused to a central nucleotide binding domain and collectively called the NB-ARC domain. The role of NB-ARC domain containing receptor kinases related to SE, e.g., SERK, was reported in several plant species [6].

Furthermore, Magnani et al. [59] reported in *Arabidopsis thaliana* that EC shut off biochemical pathways related to sugar metabolism and activate the transcriptional machinery. Our results are in agreement with this statement since no TCA cycle activity was present in EC. However, TCA cycle was highly active in NEC. This supports our above hypothesis about NEC battle for survival and makes the absence of TCA metabolites a predictor of the embryogenic state. At the hormonal level, EC had increased concentrations of ABA, ABAGE, Z and ZR compared to NEC. ABA has been previously reported to play an important role in embryo development [60]. Z plays an important role in *WUSCHEL* mediated maintenance of stem cell niche in the shoot apical meristem [61].

### 3.4. Metabolic and Hormonal Profiles of the Callus Phase Are Proof of Cell Totipotency

After the groundbreaking discovery that callus can be generated artificially in vitro [62] and that the balance between two plant hormones, auxin and cytokinin, determines the state of differentiation and dedifferentiation [63], the totipotency characteristic of callus cells has been widely used in both basic research and industrial applications [64]. Our results revealed a drastic decrease in endogenous hormone levels (auxins, cytokinins and the ethylene precursor ACC) after callus formation probably reflecting that the totipotency program is now established [65]. Only an increase in ABAGE levels is noted during callus phase and mainly in embryogenic calli. This suggests that cell fate is going towards an embryo expression program because ABA has been previously reported to play an important role in embryo development [60]. However, this program is still repressed since ABA is accumulated in a glucosylated form (ABAGE). Our results also showed that the totipotent state of callus is characterized by a clear shut-off of biochemical pathways related to sugar metabolism and energy production in agreement with Magnani et al. [59] in *Arabidopsis*, and a shut-off of the photosynthesis-related activity as previously reported by Ladygin et al. [66] in *Stevia rebaudiana.* The decrease reached a minimum level for phenolic compounds as previously described in ginger (*Zingiber officinale*) by Ali et al. [67]. These findings bring the first insights on the biochemical pathways involved in the expression of totipotency in callus. Consequently, exogenous hormones play an essential role to maintain the stem cell niche [31]. Only sucrose content showed a sharp decrease followed by an increase in glucose and fructose meaning that cells are getting their energy from the exogenous sucrose as recently showed by Gautier et al. [32] in Douglas-fir (*P. menziesii)*.

In addition to the embryogenic callus, embryogenic cell clusters and well-established compact primary callus clustered together based on their metabolic profiles. Surprisingly, primary callus had more similarities with the embryogenic callus than with the initial stages of dedifferentiation. This means that this stage could already contain, in a repressed way, the program leading to the regeneration of a whole plant. This theory is in agreement with Ge et al. [68] who showed that differential molecular responses are already present in very early stages of EC formation. An observed higher nucleocytoplasmic ratio in primary callus and EC also supports this theory. Primary callus represents a true limiting element that needs improvement of culture conditions to reduce the time needed to obtain a characteristic embryogenic callus (7 months). The reliable and highly efficient redifferentiation potential of cell clusters proves that the exogenous auxin and cell density conditions have been well optimized in coffee SE in order to maintain a proliferating totipotent state without losing embryogenic capacity [37].

### 3.5. Re-Establishment of Primary and Secondary Metabolims During Redifferentiation Leading to Embryo Development

To trigger the redifferentiation process towards the development of somatic embryos, cell clusters are inoculated at a low density in a fresh medium where the exogenous auxin (2,4-D) is removed. The significant increase in endogenous IAA levels along with the accumulation of tryptophan, a precursor of IAA biosynthesis, was also highlighted by Businge et al. [28] in Norway spruce cell lines during redifferentiation. Even though the medium is highly supplemented with 6-BA during redifferentiation, Z was detected at rather low concentrations in embryos, whereas ZR levels sharply increased. The relationship between auxin and cytokinin contents is evidently decisive for embryonic development [61], as the high auxin/cytokinin ratio at maturation seems to be a prerequisite for proper formation of apical and root meristems, optimal development of root caps, hypocotyls and cotyledons [69]. Endogenous ABA which was not regulated exogenously (no ABA supply), showed significantly increased levels in globular and torpedo-shaped embryos during redifferentiation into embryos. This agrees with previous work showing a marked increase in the endogenous levels of ABA and ABAGE during maturation of larch (*Larix kaempferi*) somatic embryos [60]. In conifers, high exogenous ABA concentrations (20 μM) are needed throughout the maturation process whereas the absence, or low amounts, of ABA in the medium may lead to inhibited or aberrant development characterized by numerous malformed embryos [70].

While cell respiratory pathways (glycolysis and TCA cycle) resume their activities to produce enough energy for the formation of new early embryos, an accumulation of starch is noted in developed embryos that has been reported to contribute to the maturation process [71]. Synthesis of a number of amino acids reveals that embryos are ready to produce more complex structures, i.e., storage and polarization-related proteins [72]. Our results showed that a polyamine, putrescine, accumulated. This finding agrees with Pedroso et al. [73] who postulated that putrescine might be related to the formation and development of globular embryos in *Camellia japonica*. An accumulation of phenolic compounds is also seen during embryo development, mainly chlorogenic acids, as they are key intermediaries for lignin synthesis, in addition to their more general role in reactive oxygen species scavenging and abiotic stress tolerance [74]. Polyamines have also been involved in plant abiotic stress tolerance [75], confirming the fact that somatic embryo formation is a stress-related phenomenon. Precursors of caffeine also accumulated during redifferentiation probably meaning that caffeine would be produced in later stages. Mannitol accumulated, probably to help tolerate the osmotic stress generated by the growth medium after cell density reduction [76].

## 4. Materials and Methods

### 4.1. Tissue Culture and Sampling

An intraspecific hybrid (GPFA116) proceeding from Arabica breeding program and selected for its high cup quality, high yield and vigor, was used in this study. Somatic embryogenesis was performed in the Nestlé Research laboratories (Tours, France) based on the large-scale protocols described previously for *C. arabica* [10], and carried out in five replicates, i.e., five independent leaf introductions in February, April, June, October and December 2016 from distinct 1-year old plants obtained by SE and grown in the Nestlé Research greenhouse. Explants were then cultured in Petri dishes on T1 ‘dedifferentiation 1′ medium, i.e., Murashige and Skoog (MS) half-strength solid medium supplemented with 2.3 µM 2,4-D, 4.9 µM IBA and 9.8 µM 2-iP for 1 month before transfer on T2 ‘dedifferentiation 2′ medium, i.e., MS/2 solid medium supplemented with 4.6 µM 2,4-D and 17.8 µM 6-BA for 6 months until emergence of embryogenic calli. Petri dishes were placed at 25 °C in the dark. Embryogenic calli were then inoculated at a density of 10 g/L in Erlenmeyer flasks containing M ‘proliferation’ liquid nutritive medium, i.e., medium supplemented with 1.4 µM 2,4-D and 4.4 µM 6-BA and cultured for 4 months on shakers (120 rpm) at 25 °C in the dark, until proliferation of cell clusters. To enhance somatic embryo regeneration, cell clusters were transferred to 250-mL Erlenmeyer flasks containing 100 mL of DIF ‘redifferentiation’ liquid medium lacking 2,4-D at a 10 g/L density for 1 week then at a 1 g/L density for 8 weeks until development of torpedo-shaped embryos. Erlenmeyer flasks were placed on shakers (120 rpm) at 25 °C in the dark.

Fourteen sampled stages were chosen to cover the SE process from leaf explant to torpedo-shaped embryos development as shown in Figure 1: leaves from greenhouse plants (L1), explants during dedifferentiation [0 h (L2), 1 week (D1), 2 weeks (D2), 5 weeks (D3)], compact primary callus obtained 3 months after induction (C1), embryogenic callus obtained 7 months after induction (C2), established cell clusters obtained after 4 months in proliferation medium (C3), early regeneration of embryos from cell clusters [1 week in DIF medium without reducing cell density (R1), 24 h in DIF medium after reducing cell density (R2), 72 h (R3), 10 d (R4)], globular embryos (E1) and torpedo-shaped embryos (E2). Additionally, non-embryogenic callus (NEC) was also sampled at the same time as the embryogenic callus (C2). Approximately 2 g of fresh weight/sample/replicate were collected for metabolome and hormone content analysis. For these experiments, material was kept in liquid nitrogen then freeze-dried. For histological analysis, fresh samples were incubated in a fixative solution (1% glutaraldehyde, 2% paraformaldehyde and 1% caffeine in 0.1 M phosphate buffer pH 7.0) for 24 h at 4 °C.

### 4.2. Primary Metabolite Analysis

Primary metabolites were extracted according to Dethloff et al. [77]. Briefly, freeze-dried samples were ground in a ball mill (TissueLyser II, Qiagen, Hilden, Germany) and 30 mg of each sample were swiftly mixed with 300 μL methanol, 30 μL nonadecanoic acid methylester (CAS 1731-94-8) (2 mg/mL in chloroform) and 30 μL of ^13^C_6_-sorbitol (CAS 121067-66-1) (0.2 mg/mL in methanol). 40 μL from the polar phase were dried in a ScanVac speed vacuum concentrator (Labogene, Allerød, Denmark). Dried samples were mixed with 40 μL of methoxyamine hydrochloride (CAS 593-56-6, 40 mg/mL in pyridine (CAS 110-86-1)) and incubated for 90 min at 30 °C. Then, 70 μL of N,O-bis(trimethylsilyl)trifluoroacetamide (BSTFA, CAS 25561-30-2) and 10 μL of a C_10_, C_12_, C_15_, C_18_, C_19_, C_22_, C_28_, C_32_, and C_36_ *n*-alkane mixture were added for retention index calibration [78]. The mixture was subsequently incubated for additional 30 min at 37 °C to complete the reaction. Sample aliquots of 1 μL were injected in splitless and split 1/30 mode using an Agilent 6890 injector system set to 250 °C (Santa Clara, CA, USA). Initial oven temperature was maintained at 70 °C for 1 min, then raised to 350 °C at 9 °C/min and kept at 350 °C for 5 min. Helium carrier gas flow was set to 0.6 mL/min. All samples were run on a gas chromatography-electron impact ionization-time of flight/mass spectrometry (GC–EI–TOF/MS) instrument with an Agilent 6890N gas chromatograph and a LECO Pegasus III TOF mass spectrometer using the manufacturer’s ChromaTOF software for acquisition and baseline correction (version 2.32, LECO, St. Joseph, MI, USA). Data processing and peak identity annotation was manually performed using the TagFinder visualization tool for mass spectral matching of time groups and clusters [79]. Mass spectral features were matched to the mass spectra and retention time indices of authenticated reference metabolites from the Golm Metabolome Database [80]. Criteria for manually supervised metabolite annotation were the presence of at least 3 specific mass fragments per compound and a retention index deviation <1.0% [81]. Values obtained were normalized with internal standard (^13^C_6_-sorbitol) and DW. This experiment was carried out in 5 replicates. Metabolites showing a coefficient of variation >80% between replicates in at least 3 stages of the SE process were removed.

### 4.3. Secondary Metabolite Analysis

The freeze dried samples were ground into fine powder in a ball mill (TissueLyser II, Qiagen) and extracted by stirring (225 rpm, Rotamax 120, Heidolph, Schwabach, Germany) 15 mg of each collected sample in 600 µL of methanol (MeOH)/H_2_O (80:20, *v*/*v*) at 4 °C, in the dark, for 3 h. After centrifugation (10 min, 8 °C, 3500 rpm), the methanolic extract was collected and filtered (0.25 µm porosity, Interchim, Montluçon, France) before analysis. Each extraction was carried out in triplicate.

Quantitative analyses were carried out on a high-performance liquid chromatography (HPLC) system (Shimadzu LC-20, Tokyo, Japan) equipped with a binary pump, a photodiode array detector (DAD) and an Eclipse XDB C18 (3.5 µm) column (100 mm × 4.6 mm, Agilent). Extracts (10 µL) were analyzed at a flow rate of 0.6 mL/min using an elution system composed of solvents B (H_2_O/MeOH/acetic acid, 5:90:5 *v*/*v*/*v*) and A (water/acetic acid, 98:2, *v*/*v*) mixed as described in Campa et al. [82]. Parallel analyses were performed in triplicate on pure standard solutions of theobromine, caffeine, mangiferin, 3,4-, 3,5- and 4,5-O-diCQA, 5-CQA and caffeic acid from Sigma-Aldrich (St Quentin Fallavier, France), glycosylated kaempferol and quercetin, rutin, (+)-catechin, (−)-epicatechin and epigallocatechin from Extrasynthese (Lyon, France) at 10, 25, 50, and 75 µg/mL. Quantification of 3-, 4- and 5-CQA, FQA and 3,4-, 3,5- and 4,5-diCQA was undertaken at 320 nm, caffeine and catechin derivatives at 280 nm, and mangiferin, kaempferol and quercetin derivatives at 360 nm. Concentration was expressed in mg/g dry weight by comparison with the standard curves established with respective standards. For 3-CQA, 4-CQA and FQA, content was calculated taking into account the 5-CQA standard curve. Each coffee leaf powder sample was extracted and analyzed twice. Values obtained were normalized with DW.

### 4.4. Hormone Content Analysis

Quantification of widely reported phytohormones, i.e., ABA and ABA catabolites, auxins, cytokinins, gibberellins and the ethylene precursor ACC, was conducted at the National Research Council of Canada (Saskatoon, SK, Canada) by ultra-performance liquid chromatography-electrospray tandem mass spectrometry (UPLC-ESI–MS/MS) [83] using deuterium labeled internal standards. The procedure for the hormone profiling analysis was previously described in detail [84,85]. This experiment was carried out in triplicate. Results were expressed as ng/g DW. Hormones showing a coefficient of variation >80% between replicates in at least 3 of the 14 stages of the SE process were removed.

### 4.5. Starch Content Assay

The starch content of 20 mg of freeze-dried powder was determined using the total starch kit GOPOD (d-glucose, k-Gluc, Megazyme International, Wicklow, Ireland). After elimination of soluble sugars and of the soluble products of starch degradation, the residue was successively hydrolysed into glucose units with α-amylase and amyloglucosidase. The resulting d-glucose was then degraded with glucose oxidase and the resulting hydrogen peroxide quantified by spectrophotometry at 510 nm after a last enzymatic reaction. Values obtained were normalized with DW.

### 4.6. Histological Analysis and Cell Imaging

Fixated samples were dehydrated by successive immersions of 1 h-duration each in graded solvent series of ethanol, from 50% to 100%. Samples were then embedded in Technovit 7100 resin (Kulzer, Werheim, Germany) according to the manufacturer’s instructions. The cross-sections (3.5 μm), obtained with a Leica RM2255 microtome (Leica Microsystems, Wetzlar, Germany), were oxidized in 1% periodic acid (5 min), washed with distilled water and stained in the dark for 10 min with Schiff reagent (ref. 3952016, Sigma Aldrich). After washing, sections were stained with Naphthol Blue Black [1 g in 7% (*v*/*v*) (ref. 1.01167, Sigma Aldrich)] for 5 min at 60 °C, treated with 7% acetic acid and finally dried for 15 min at 60 °C. A total of 9 sections per time point were analysed. Images were obtained under a Nikon Eclipse Ni-E microscope (Nikon Instruments Inc., Melville, NY, USA) with two objectives, 20× and 40×, after acquisition using NIS software (Nikon Instruments Inc.). Images were analyzed using Image J 1.47v software.

Chlorogenic acid localization in living cells was carried out according to Talamond et al. [40]. Fresh samples were mounted on a glass slide in a drop of water and observed using a Zeiss LSM880 multiphoton microscope (Zeiss, Jena, Germany), equipped with a Chameleon Ultra II laser (Coherent, Santa Clara, CA, USA) and with an 20x Plan Apo 1.0 NA objective. Excitation wavelength was set to 720 nm and emission was observed with a band-pass filter, 386-502 nm (blue). Image acquisition was performed using Zen software (Zeiss). The presence and accumulation of total chlorogenic acids during embryo redifferentiation was confirmed by multiphoton microscopy combined with emission spectral analysis of total chlorogenic acids (Figure S2). After obtaining the spectral acquisitions, the LinearUnmixing function (Zen software) was executed to separate, pixel by pixel, the mixed signals of defined pure autofluorescent compounds (chlorogenic acids and chlorophyll), using the entire emission spectrum of each compound, plus a residual channel. In the residual channel, the intensity values represented the difference between the acquired spectral data and the fitted linear combination of the reference spectra.

### 4.7. Statistical Analysis

All analyses were carried out with R [86] using the default functions. First, a Principal Component Analysis (PCA) was carried out to verify homogeneity of replicates using the prcomp function (package stats) (Figure S1). For each primary or secondary metabolite, normalized values obtained at each stage were transformed to Z-scores by subtracting the mean of the values obtained at all collected stages and dividing it by the standard deviation. The Z-score transformation provides a way of standardizing data across a wide range of experiments [87]. A Z-score equal to 1 is one standard deviation above the mean. Heatmaps of Z-scores were generated using the ComplexHeatmap package [88]. The same data were used for the analysis of hierarchical clustering. Hierarchical clustering was performed with the pvclust package [89] using Pearson’s correlation coefficient [90]. Cluster probabilities were calculated via a multiscale bootstrap with a total of 1000 iterations. A cluster probability is a percentage which indicates how strong the cluster is supported by data. One-way ANOVA was used to calculate any significant differences (at *P* = 0.05) in cell measurements between the 14 sampled stages of the SE process, followed by a Tukey test as post-hoc. Similarly, a Student *t*-test was used to compare cell measurements in embryogenic (EC) and non-embryogenic calli (NEC). Normality of distribution and equality of variance were verified prior to these tests. Concerning hormone content, first, the mean of values of the different sampled stages was calculated for each developmental phase then a Kruskal-Wallis test was used to calculate any significant differences (at *P* = 0.05) in mean hormone levels between phases, followed by a Dunn test as a post-hoc (dunn.test package). A non-parametric two-by-two Wilcoxon mean comparison test was used to assess significant differences (at *P* = 0.05) in hormone content between the four phase switches and between EC and NEC. Highlighted metabolic pathways over the four switches were identified based on an enrichment analysis using Metaboanalyst [91] in significantly over-accumulated and under-accumulated metabolites (Z-score >1 or <−1).

## 5. Conclusions

This first global analysis of SE metabolomics, applied on coffee, one of themodel tree species, showed that metabolomics provides a powerful approach to investigate global metabolite patterns during the SE process. This approach helped to clearly divide the 14 sampled stages of the SE process into five important phases (Leaf, Dedifferentiation, Callus, Redifferentiation and Embryo) leading to the identification of four key developmental phase switches, which are strategic for the whole biological efficiency of embryo regeneration. This global analysis showed that metabolites provide a specific signature for each developmental phase and bring us rich information on cell fate, as some of the sampled stages were undistinguishable by conventional morphological and histological studies (e.g., the first dedifferentiation and redifferentiation events). A number of metabolic markers of the embryogenic state were identified and can be directly used as targets to pilot the optimization of the composition of the culture media. This global metabolomics analysis brings first insights to unravel the metabolic and hormonal mechanisms taking place during SE and will be combined with a transcriptomic approach we are carrying on the same sampled stages in order to give a much clearer understanding of the intimate mechanisms governing totipotency and embryogenesis. This study represents a starting point for optimizing coffee SE protocols in a rational way, putting an end to thirty years of empirical research. These findings should be informative and useful to a wide range of plant species, offering unprecedented perspectives in plant micropropagation.

## Figures and Tables

**Table 1:** Characteristics of the main represented cell types in each of the 15 key sampled stages of the Arabica SE process.

Cell Type	Stage	Cell Length (µm)	Cell Width (µm)	W/L Ratio	Cytoplasm Size (µm^2^)	Nucleus Size (µm^2^)	N/C Ratio	Cell Division	Starch	Proteins
Palisade mesophyll cells	L1	41.5 ± 1.7 ^a^	13.9 ± 2.5 ^a^	0.33 ± 0.04 ^a^	430 ± 93 ^a^	66.5 ± 25 ^a^	0.17 ± 0.02 ^a^	-	+	+
Palisade mesophyll cells	L2	38.2 ± 4.1 ^a^	13.0 ± 2.0 ^a^	0.35 ± 0.01 ^a^	440 ± 99 ^a^	59.1 ± 17 ^a^	0.19 ± 0.03 ^a^	-	+	+
Dedifferentiated cells	D1	33.9 ± 2.5 ^a^	22.8 ± 4.0 ^b^	0.67 ± 0.08 ^b^	647 ± 71 ^b^	56.8 ± 7.4 ^a^	0.09 ± 0.02 ^a^	+++	+	+
Dedifferentiated cells	D2	32.7 ± 1.8 ^a^	22.4 ± 0.7 ^b^	0.69 ± 0.02 ^b^	681 ± 44 ^b^	155 ± 18.3 ^b^	0.24 ± 0.04 ^b^	+++	+	+
Dedifferentiated cells	D3	35.8 ± 2.6 ^a^	27.2 ± 5.1 ^b^	0.76 ± 0.11 ^b^	695 ± 91 ^b^	55.4 ± 11.3 ^a^	0.08 ± 0.03 ^a^	+++	++	+
Primary callus cells	C1	35.9 ± 2.1 ^a^	30.6 ± 2.6 ^b^	0.85 ± 0.02 ^b^	784 ± 56 ^b^	114 ± 25.3 ^b^	0.19 ± 0.03 ^a^	+	++	+
Embryogenic cells	C2	20.2 ± 1.3 ^b^	14.7 ± 0.5 ^a^	0.73 ± 0.03 ^b^	214 ± 18 ^c^	70.7 ± 5.1 ^a^	0.33 ± 0.02 ^b^	++	+++	+++
Embryogenic cell clusters	C3	23.9 ± 1.0 ^b^	12.7 ± 1.1 ^a^	0.54 ± 0.06 ^a^	223 ± 15 ^c^	70.1 ± 10.5 ^a^	0.31 ± 0.03 ^b^	++++	+++	+++
Pro-embryogenic masses	R1	19.5 ± 2.7 ^b^	12.1 ± 0.5 ^a^	0.62 ± 0.04 ^b^	144 ± 15 ^c^	66.3 ± 8.4 ^a^	0.45 ± 0.07 ^b^	+++	+++	+++
Pro-embryogenic masses	R2	20.2 ± 1.8 ^b^	13.9 ± 1.5 ^a^	0.69 ± 0.54 ^b^	202 ± 23 ^c^	65.2 ± 10.8 ^a^	0.32 ± 0.02 ^b^	+++	+++	+++
Pro-embryogenic masses	R3	18.8 ± 1.3 ^b^	12.5 ± 0.3 ^a^	0.67 ± 0.03 ^b^	165 ± 13 ^c^	69.0 ± 15.7 ^a^	0.41 ± 0.07 ^b^	+++	+++	+++
Pro-embryogenic masses	R4	26.7 ± 1.4 ^b^	16.5 ± 1.6 ^a^	0.70 ± 0.07 ^b^	315 ± 26 ^c^	98.7 ± 6.6 ^a^	0.31 ± 0.03 ^b^	+++	+++	+++
Embryonic cells	E1	22.5 ± 1.0 ^b^	14.5 ± 1.6 ^a^	0.64 ± 0.04 ^b^	236 ± 24 ^c^	24.2 ± 4.2 ^c^	0.10 ± 0.01 ^a^	+++	+	+++
Embryonic cells	E2	42.0 ± 1.5 ^a^	31.9 ± 0.7 ^b^	0.76 ± 0.04 ^b^	943 ± 55^d^	88.5 ± 10.3 ^a^	0.09 ± 0.01 ^a^	+++	+	++

Values represent mean ± SD of 30 cells analyzed for each sampled stage. Stages correspond to: leaves from greenhouse plants (L1), explants during dedifferentiation [0 h (L2), 1 week (D1), 2 weeks (D2), 5 weeks (D3)], compact primary callus obtained 3 months after induction (C1), embryogenic callus obtained 7 months after induction (C2), established cell clusters obtained after 4 months in liquid proliferation medium (C3), pro-embryogenic masses [1 week in redifferentiation medium after auxin withdrawal (R1), 24 h in redifferentiation medium after reducing cell density (R2), 72 h (R3), 10 days (R4)], globular embryos (E1) and torpedo-shaped embryos (E2). W/L ratio corresponds to the mean of ratios between width and length for each cell. N/C ratio corresponds to the mean of ratios between nucleus and cytoplasm for each cell (nucleus-to-cytoplasm ratio). Cell division activity was estimated based on the number of cells in telophase. Starch granules were evidenced by the Schiff reagent and soluble proteins were evidenced by Naphthol Blue Black. Differences between the sampled stages were analysed with a one-way ANOVA test. Data followed by different letters in a same column are significantly different according to the Tukey post-hoc test (*P* < 0.05).

**Table 2:** Significantly over-accumulated metabolites in the five developmental phases forming the Arabica SE process.

Metabolic Pathways	Developmental Phases of Coffee Somatic Embryogenesis				
	Leaf	Dedifferentiation	Callus	Redifferentiation	Embryo
TCA cycle		Aconitic acid		Aconitic acid	Fumaric acid
		Citric acid			Malic acid
		Fumaric acid			Succinic acid
		Glutaric acid, 2-oxo-			
		Malic acid			
		Succinic acid			
Sugar metabolism		Glucose-6-phosphate	Glucose	Glucose-6-phosphate	Glucose-6-phosphate
		Mannose-6-phosphate	Fructose	Fructose-6-phosphate	Fructose-6-phosphate
		Maltose	Maltotriose	Mannose-6-phosphate	Mannose-6-phosphate
		Sucrose	Ribose	Ribose	Starch
Amino acid metabolism	Alanine, beta-	Alanine, beta-	Glycine	Leucine	Alanine
	Aspartic acid	Aspartic acid	Methionine	Lysine	Glycine
		Asparagine	Proline	Proline	Leucine
		Glycine			Lysine
		Glutamine			Methionine
		Valine			Proline
					Glutamate
Fatty acid metabolism		Hexadecanoic acid	Glycerol	Glycerol-3-phosphate	Glycerol
		Octadecanoic acid			Glycerol-3-phosphate
		Glyceric acid			
Polyols			Inositol, myo-	Inositol, myo-	Inositol, myo-
					Mannitol
N-compounds					Putrescine
Ascorbate and aldarate metabolism	Galactonic acid	Arabinonic acid			
	Threonic acid	Galactaric acid			
		Galactonic acid			
Shikimate pathway	Quinic acid	Quinic acid		Tryptophan	Tryptophan
	Shikimic acid	Phenylalanine		Caffeic acid	
		Caffeic acid		Benzoic acid	
Alkaloid precursors		Nicotinic acid		Guanosine	
		Xanthosine		Xanthosine	
				Inosine	
Alkaloids	Trigonelline				
	Theobromine				
	Caffeine				
Phenolic compounds	Mangiferin				3-CQA
	Catechin				3,5-diCQA
	Epicatechin				4-CQA
	3-CQA				4,5-diCQA
	3,4-diCQA				5-CQA
	4-CQA				FQA
	FQA				

All primary and secondary metabolites displaying significant increased values from their respective means across the 14 stages (Z-score > 1) were listed in this table and were classified according to Kyoto Encyclopedia of Genes and Genomes (KEGG) database of metabolic pathways. CQA: caffeoylquinic acid, FQA: feruloylquinic acid, TCA: tricarboxylic acid.

**Table 3:** Significantly under-accumulated metabolites in the five developmental phases forming the Arabica SE process.

Metabolic Pathways	Developmental Phases of Coffee Somatic Embryogenesis				
	Leaf	Dedifferentiation	Callus	Redifferentiation	Embryo
TCA cycle			Fumaric acid		
			Glutaric acid, 2-oxo-		
			Malic acid		
Sugar metabolism	Fructose-6-phosphate	Glucose	Fructose-6-phosphate		Fructose
	Glucose-6-phosphate	Fructose	Glucose-6-phosphate		
	Mannose-6-phosphate	Starch	Mannose-6-phosphate		
	Fructose		Sucrose		
	Ribose				
Amino acid metabolism	Leucine	Lysine	Leucine	Valine	
	Lysine		Lysine		
			Valine		
Ascorbate and aldarate metabolism			Arabinonic acid		
			Galactonic acid		
			Threonic acid		
Polyols	Inositol, myo-				

All primary and secondary metabolites displaying significant decreased values from their respective means across the 14 stages (Z-score < −1) were listed in this table and were classified according to KEGG database of metabolic pathways. TCA: tricarboxylic acid.

**Table 4:** Hormone dynamics during the five main developmental phases of the Arabica SE process.

Hormones	Developmental Phases of Coffee Somatic Embryogenesis				
	Leaf >	Dedifferentiation >	Callus >	Redifferentiation >	Embryo
ABA	583 ± 72 ^a^	113 ± 16 ^b^	74 ± 12 ^b^	70 ± 12 ^b^	600 ± 55 ^a^
ABAGE	243 ± 45 ^a^	121 ± 36 ^a^	675 ± 41 ^b^	671 ± 77 ^b^	1230 ± 103 ^c^
ACC	1281 ± 173 ^a^	19491 ± 447 ^b^	3063 ± 272 ^a^	3179 ± 193 ^a^	3065 ± 169 ^a^
IAA	34 ± 6 ^a^	182 ± 27 ^b^	17 ± 7 ^a^	60 ± 8 ^c^	81 ± 19 ^c^
IBA	N.D. ^a^	4345 ± 385 ^b^	N.D. ^a^	N.D. ^a^	N.D. ^a^
iP	N.D. ^a^	4557 ± 441 ^b^	N.D. ^a^	N.D. ^a^	N.D. ^a^
Z	2 ± 1 ^a^	17 ± 3 ^b^	2 ± 1 ^a^	N.D. ^a^	4 ± 2 ^b^
ZR	6 ± 3 ^a^	13 ± 3 ^a^	22 ± 4 ^b^	15 ± 5 ^a^	60 ± 6 ^c^

Values represent the mean of hormone levels, expressed in ng/g DW, in the sampled stages forming each of the five phases (Leaf, Dedifferentiation, Callus, Redifferentiation and Embryo) in three independent replicates ± SD. For each hormone, differences in levels between phases were analysed with a Kruskal-Wallis test. Data followed by different letters are significantly different according to Dunn post-hoc test (*P* < 0.05). Blue color corresponds to an increase in hormone levels between a phase and the previous one (column on the left) while grey color corresponds to a decrease in hormone levels. N.D. stands for not detected. ABA: *cis*-abscisic acid, ABAGE: ABA glucose ester, IAA: indole-3-acetic acid, IBA: indole 3-butyric acid, iP: isopentenyladenine, Z: zeatin (*cis*- and *trans*-), ZR: zeatin riboside (*cis*- and *trans*-).

**Table 5:** Characteristics of the mainly represented cell populations in embryogenic (EC) and non-embryogenic calli (NEC).

Cell Type	Stage	Cell Length (µm)	Cell Width (µm)	W/L Ratio	Cytoplasm Size (µm^2^)	Nucleus Size (µm^2^)	N/C Ratio	Cell Division	Starch	Proteins
Embryogenic cells	EC	20.2 ± 1.3 ^a^	14.7 ± 0.5 ^a^	0.73 ± 0.03 ^a^	214 ± 18.0 ^a^	70.7 ± 5.1 ^a^	0.33 ± 0.02 ^a^	++	++	+++
Non-embryogenic cells	NEC	69.7 ± 10.7 ^b^	41.3 ± 1.6 ^b^	0.60 ± 0.08 ^a^	2081 ± 386 ^b^	364 ± 53 ^b^	0.17 ± 0.02 ^b^	+++	+++	+

Values represent mean ± SD of 30 cells analyzed for each sampled stage. W/L ratio corresponds to the mean of ratios between each cell width and length. N/C ratio corresponds to the mean of ratios between each cell nucleus and cell size. Cell division activity was estimated on the basis of the number of cells in telophase. Starch reserves and proteins were evidenced by the Schiff reagent and Naphthol Blue Black, respectively. Data followed by different letters are significantly different according to Student *t*-test (*P* < 0.05).

**Table 6:** Over-accumulated and under-accumulated metabolites in embryogenic and non-embryogenic calli.

Metabolic Pathways	Over-Accumulated Metabolites		Under-Accumulated Metabolites	
	Embryogenic Callus	Non-Embryogenic Callus	Embryogenic Callus	Non-Embryogenic Callus
TCA cycle		Citric acid	Fumaric acid	
		Glutaric acid, 2-oxo-	Glutaric acid, 2-oxo-	
		Malic acid	Malic acid	
		Succinic acid		
Sugar metabolism	Glucose	Starch	Fructose-6-phosphate	Fructose-6-phosphate
	Fructose	Ribose	Glucose-6-phosphate	Glucose-6-phosphate
	Maltotriose		Mannose-6-phosphate	Mannose-6-phosphate
	Ribose			
Amino acid metabolism	Glycine	Alanine, beta-	Leucine	Proline
	Methionine	Glycine	Valine	Valine
	Proline	Leucine		
		Methionine		
Fatty acid metabolism	Glycerol	Glycerol		
Polyols	Inositol, myo-		Inositol, myo-	
Ascorbate and aldarate metabolism			Arabinonic acid	Arabinonic acid
			Threonic acid	Threonic acid
			Galactonic acid	

All primary and secondary metabolites displaying significant increased values (Z-score > 1) and decreased values (Z-score < −1) between embryogenic callus and non-embryogenic callus, were listed in this table and were classified according to KEGG database of metabolic pathways. TCA: tricarboxylic acid.

**Table 7:** Hormone levels in embryogenic and non-embryogenic calli.

Hormones	Embryogenic Callus	Non-Embryogenic Callus
ABA	130 ± 14 ^a^	8 ± 2 ^b^
ABAGE	1200 ± 84 ^a^	24 ± 11 ^b^
ACC	3074 ± 96 ^a^	3016 ± 161 ^a^
IAA	25 ± 6 ^a^	29 ± 3 ^a^
IBA	N.D. ^a^	N.D. ^a^
iP	N.D. ^a^	N.D. ^a^
Z	2 ± 1 ^a^	N.D. ^b^
ZR	40 ± 5 ^a^	10 ± 3 ^b^

Values represent the mean ± SD of 3 independent replicates, expressed in ng/g DW. Non-parametric two-by-two Wilcoxon mean comparison test was used to calculate any significant differences in hormone levels between embryogenic and non-embryogenic calli. Data followed by different letters are significantly different (*P* < 0.05). Grey color corresponds to a decrease in hormone levels in non-embryogenic calli compared to embryogenic-calli. N.D. stands for not detected. ABA: *cis*-abscisic acid, ABAGE: ABA glucose ester, IAA: indole-3-acetic acid, IBA: indole 3-butyric acid, iP: isopentenyladenine, Z: zeatin (*cis*- and *trans*-), ZR: zeatin riboside (*cis*- and *trans*-).

## Supplementary Materials

Supplementary materials can be found at https://www.mdpi.com/1422-0067/20/19/4665/s1.

## Abbreviations

2,4-D	2,4-Dichlorophenoxyacetic acid
2-iP	N^6^-(2-Isopentenyl) adenine
6-BA	6-Benzylaminopurine
ABA	*cis*-Abscisic acid
ABAGE	ABA glucose ester
ACC	1-Aminocyclopropane-1-carboxylic acid
BSTFA	N,O-Bis(trimethylsilyl)trifluoroacetamide
CQA	Caffeoylquinic acid
DAD	Photodiode array detector
DW	Dry weight
EC	Embryogenic callus
FQA	Feruloylquinic acid
GC–EI–TOF/MS	Gas chromatography-electron impact ionization-time of flight/mass spectrometry
GC-MS	Gas chromatography–mass spectrometry
HPLC	High-performance liquid chromatography
IAA	Indole-3-acetic acid
IBA	Indole 3-butyric acid
iP	Isopentenyladenine
KEGG	Kyoto Encyclopedia of Genes and Genomes
LC-MS	Liquid chromatography–mass spectrometry
MeOH	Methanol
MS	Murashige and Skoog
N/C ratio	Nucleocytoplasmic ratio
NEC	Non-embryogenic callus
PCA	Principal Component Analysis
PEMs	Pro-embryogenic masses
SE	Somatic embryogenesis
TCA	Tricarboxylic acid
UPLC-ESI–MS/MS	Ultra-performance liquid chromatography-electrospray tandem mass spectrometry
W/L ratio	Width-to-length ratio
Z	Zeatin (*cis*- and *trans*-)
ZR	Zeatin riboside (*cis*- and *trans*-)
