# Supplementary material for: Unravelling the Metabolic and Hormonal Machinery During Key Steps of Somatic Embryogenesis: A Case Study in Coffee
Source: Int J Mol Sci. 2019 Sep 20;20(19):4665. doi: 10.3390/ijms20194665 (PMC6802359; doi:10.3390/ijms20194665)
Supplement: Supplementary file 1 [file ijms-20-04665-s001.pdf]

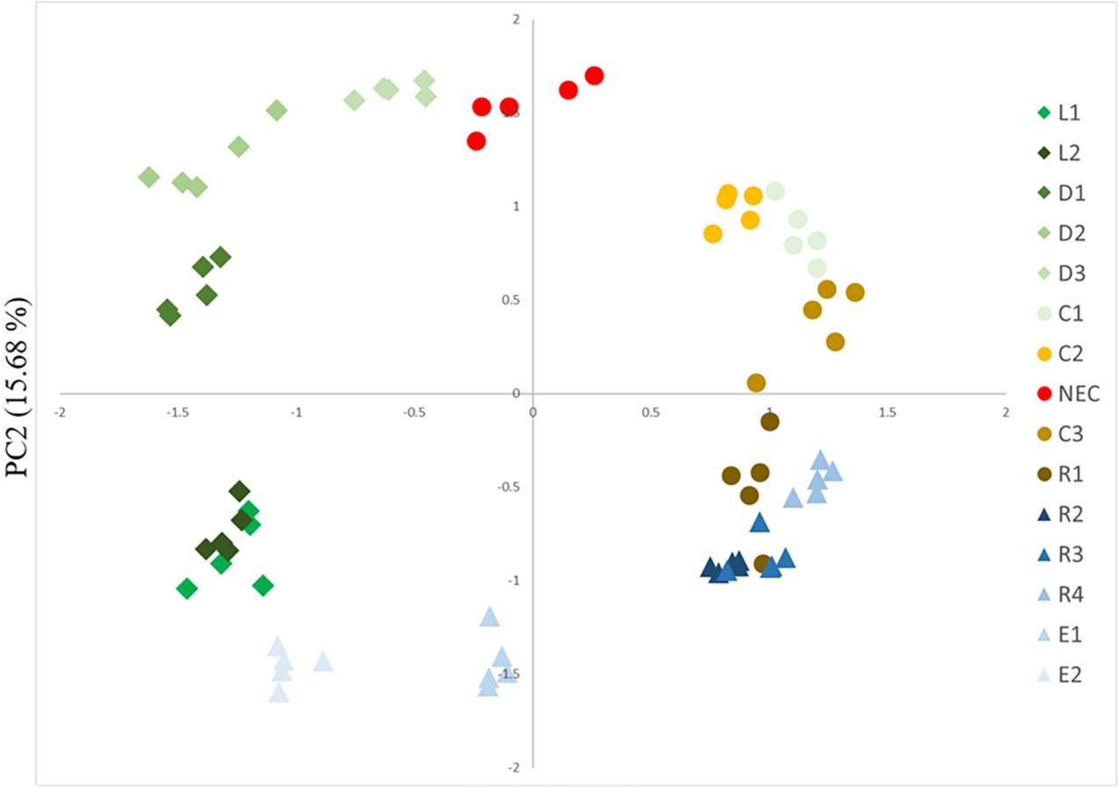

Figure S1. Assessment of homogeneity of metabolic replicates in 15 key sampled stages of the somatic embryogenesis process. A Principal Component Analysis (PCA) was carried out to verify homogeneity of 5 independent replicates; i.e. 5 independent leaf introductions in February, April, June, October and December 2016. Samples correspond to : Leaves from greenhouse plants (L1), explants during dedifferentiation [0 h (L2), 1 week (D1), 2 weeks (D2), 5 weeks (D3)], compact primary callus obtained 3 months after induction (C1), embryogenic callus obtained 7 months after induction (C2), established cell clusters obtained after 4 months in proliferation medium (C3), early regeneration of embryos from cell clusters [1 week in DIF medium without reducing cell density (R1), 24 h in DIF medium after reducing cell density (R2), 72 h (R3), 10 d (R4)], globular embryos (E1) and torpedo-shaped embryos (E2). Non-embryogenic callus (NEC) was also sampled at the same time as the embryogenic callus (C2).

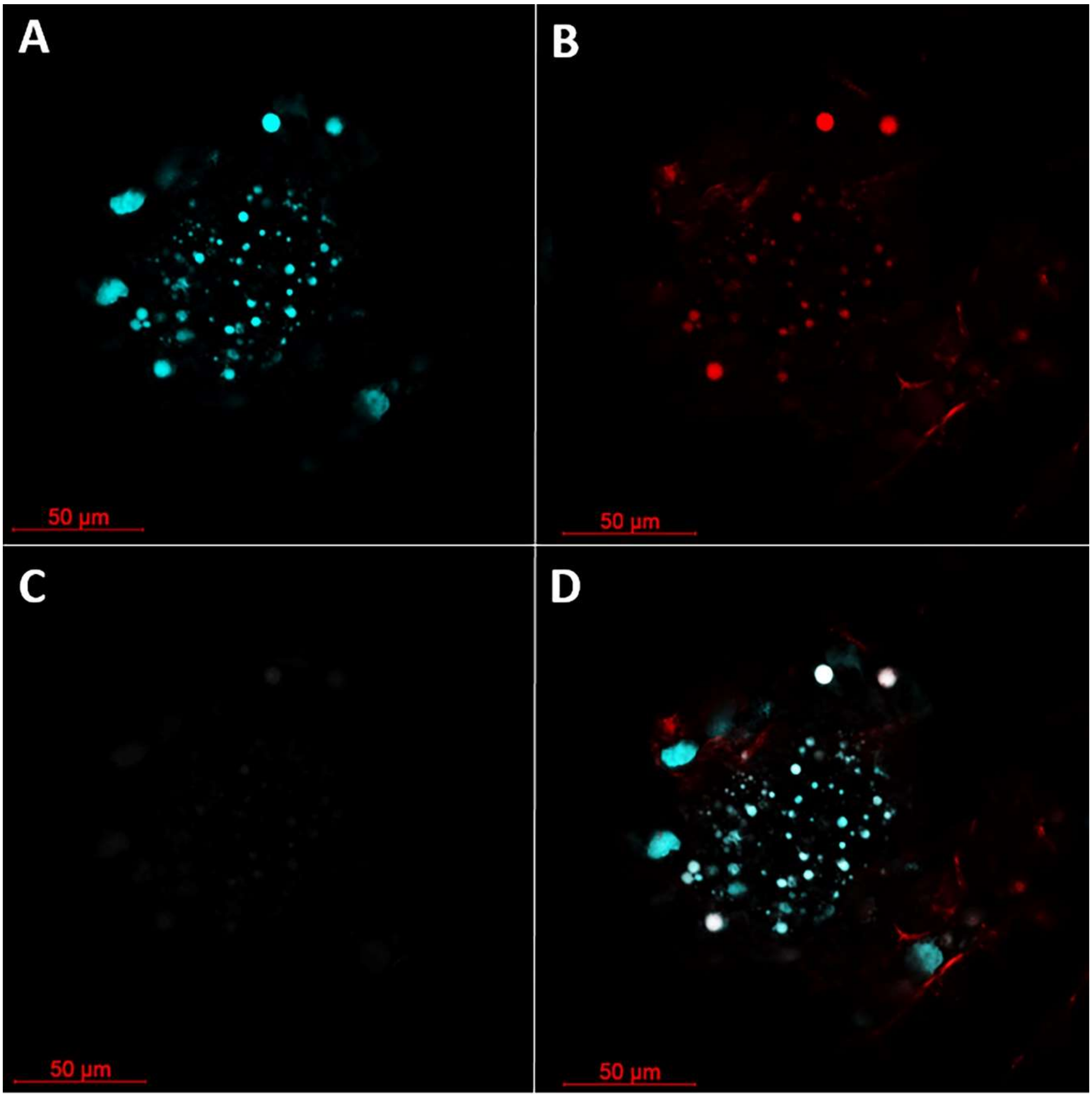

Figure S2. Validation of the presence of total chlorogenic acids in Arabica globular-shaped embryos by multiphoton microscopy combined with emission spectral analysis. After obtaining the spectral acquisitions, the LinearUnmixing function (Zen software) was executed to separate, pixel by pixel, the mixed signals of defined pure autofluorescent compounds (chlorogenic acids (**A**) and chlorophyll (**B**)), using the entire emission spectrum of each compound, plus a residual channel (**C**). An overlay image of the three channels was finally generated (**D**).

Table S1. List of primary and secondary metabolites detected over the 15 sampled stages of the Arabica somatic embryogenesis process.

Metabolite classification	Metabolites detected
**Primary metabolites**	2-Imidazolidone-4-carboxylic acid
	Aconitic acid, cis-
	Adenosine-5-monophosphate/Guanosine-5-monophosphate
	Alanine
	Alanine, beta-
	Arabinonic acid
	Arabinose
	Asparagine
	Aspartic acid
	Benzoic acid
	Benzoic acid, 4-hydroxy-
	Benzylalcohol
	Boric acid
	Butanoic acid, 4-hydroxy-
	Caffeic acid, trans-
	Citric acid
	Erythritol
	Erythronic acid
	Ethanolamine
	Ethanolaminephosphate
	Fructose
	Fructose-1-phosphate
	Fructose-6-phosphate
	Fumaric acid
	Furan-2-carboxylic acid
	Galactaric acid
	Galactinol
	Galactonic acid
	Gentiobiose
	Gluconic acid
	Glucose
	Glucose-6-phosphate
	Glutaric acid, 2-oxo-
	Glutaric acid, 3-hydroxy-3-methyl-
	Glyceric acid
	Glyceric acid-3-phosphate
	Glycerol
	Glycerol-3-phosphate
	Glycerophosphoglycerol
	Glycine
	Glycolic acid
	Guanosine
	Hexadecanoic acid
	Hydroquinone
	Inosine
	Inositol, myo-

	Inositol-phosphate, myo-
	Isocaproic acid, 2-oxo-
	Lactic acid
	Leucine
	Lysine
	Malic acid
	Malic acid, 2-methyl-
	Malonic acid
	Maltose
	Maltotriose
	Mannitol
	Mannose-6-phosphate
	Methionine
	Nicotinic acid
	Octadecanoic acid
	Orotic acid
	Phenylalanine
	Phosphoric acid
	Phosphoric acid monomethyl ester
	Piperidine-2-carboxylic acid
	Proline
	Proline, 4-hydroxy-, cis-
	Propanoic acid, 2-amino-2-methyl-3-hydroxy-
	Purine, 6-benzylamino-, 9-beta-D-glucopyranosyl-
	Putrescine
	Pyroglutamic acid + Glutamic acid + Glutamine
	Quinic acid
	Raffinose
	Ribitol
	Ribonic acid
	Ribose
	Saccharic acid
	Salicylic acid
	Shikimic acid
	Sorbitol
	Starch
	Succinic acid
	Sucrose
	Thiazole, 4-methyl-5-hydroxyethyl-
	Threonic acid
	Trigonelline
	Tryptophan
	Uridine
	Valine
	Xanthosine
	Xylose
**Secondary metabolites**	3-Caffeoylquinic acid
	3,4-diCaffeoylquinic acid

3,5-diCaffeoylquinic acid
4-Caffeoylquinic acid
4,5-diCaffeoylquinic acid
5-Caffeoylquinic acid
Caffeine
Catechin
Epicatechin
Feruloylquinic acid
Mangiferin
Theobromine
